# Randomised Trial of Chloroquine/Sulphadoxine-Pyrimethamine in Gambian Children with Malaria: Impact against Multidrug-Resistant P. falciparum

**DOI:** 10.1371/journal.pctr.0010014

**Published:** 2006-07-21

**Authors:** Samuel Dunyo, Rosalynn Ord, Rachel Hallett, Musa Jawara, Gijs Walraven, Eduardo Mesa, Rosalind Coleman, Maimuna Sowe, Neal Alexander, Geoffrey A. T Targett, Margaret Pinder, Colin J Sutherland

**Affiliations:** 1 Farafenni Field Station, Medical Research Council Laboratories, Fajara, The Gambia; 2 Immunology Unit and Infectious Disease Epidemiology Unit, Department of Infectious and Tropical Diseases, London School of Hygiene and Tropical Medicine, London, United Kingdom

## Abstract

**Objectives:**

In the Gambia, the combination of chloroquine (CQ) and sulphadoxine-pyrimethamine (SP) has replaced CQ monotherapy for treatment of malaria caused by Plasmodium falciparum. We measured the efficacy of the combination CQ/SP, and the prevalence of parasites carrying alleles associated with resistance to CQ or SP.

**Design:**

We conducted a single-blind, randomised, controlled trial to compare the efficacy of CQ/SP to that of SP or CQ alone.

**Setting:**

The study took place in the town of Farafenni and surrounding villages in the Gambia.

**Participants:**

Participants were **c**hildren aged 12 mo to 10 y presenting as outpatients with uncomplicated P. falciparum malaria.

**Interventions:**

500 children were randomised to receive CQ, SP, or CQ/SP as supervised treatment and actively followed over 28 d.

**Outcome Measures:**

Primary outcome was parasitaemia at any time during follow-up. Secondary outcomes were PCR-confirmed recrudescent infections among treatment failures, and clinical failure requiring rescue medication by day 28. Pretreatment parasite isolates from 161 patients were tested for the presence of resistance-associated genetic markers.

**Results:**

The prevalence of parasitological failure by day 28 for the CQ group was 60.3%, compared to 17.6% for SP (odds ratio [OR], 0.106; 95% confidence interval [CI], 0.057–0.194; *p* < 0.001) and 13.9% for CQ/SP (OR versus CQ, 0.140; 95% CI, 0.078–0.250; *p* < 0.001). There was no difference between the SP and CQ/SP groups (OR, 1.324; 95% CI, 0.705–2.50). The projected prevalence of PCR-corrected treatment failure was 30.2, 6.06, and 3.94% in the CQ, SP, and CQ/SP groups, respectively. The *pfdhfr*-triple mutant and *pfdhps*-437G mutation were common, with prevalences of 67.4 and 51.2%, respectively. Pretreatment carriage of *pfdhps-*437G and of multidrug-resistant parasite genotypes was associated with treatment failure in the SP group, but not in the CQ or CQ/SP groups.

**Conclusions:**

The combination of CQ/SP was an efficacious treatment for uncomplicated malaria in Gambian children in this study, but the frequent occurrence of multidrug-resistant parasites suggests that this observed efficacy is not sustainable.

## Editorial Commentary

**Background:** Throughout Africa, the readily available and cheap antimalarial drug, chloroquine, has been failing as a good first-line treatment for uncomplicated malaria. This is mainly due to the malaria parasite becoming resistant to treatment. Many African countries are now adopting newer artemisinin-based combination therapies as first-line treatment. However other countries have put interim solutions in place before introducing artemisinin-based drugs. The combination of chloroquine and sulfadoxine-pyrimethamine was officially adopted as first-line therapy in the Gambia in 2004. However, little data exists on the efficacy of this combination in that setting. It is also not known whether parasites resistant to sulfadoxine-pyrimethamine, as well as chloroquine, are common in the Gambia. In this trial, conducted in a rural town in the Gambia in 2001, the researchers randomized 500 young children presenting with uncomplicated P. falciparum malaria to receive either chloroquine, sulfadoxine-pyrimethamine, or the combination of both treatments. The researchers then looked for the presence of parasites in the blood of treated children over 28 days of follow-up. Infections following treatment were defined as either the same infection that was originally treated (a recrudescence) or a new infection. The need for rescue medication, indicating clinical failure, was also examined.

**What the trial shows:** In this trial, both sulfadoxine-pyrimethamine and the combination of chloroquine and sulfadoxine-pyrimethamine were more effective at clearing parasites from the blood than chloroquine alone. However the combination of chloroquine and sulfadoxine-pyrimethamine appeared to be equivalent to sulfadoxine-pyrimethamine in clearing parasite infection. When the researchers distinguished parasite infections into recrudescences or new infections, they found that either treatment option containing sulfadoxine-pyrimethamine prevented a recurrence of the original infection better than treatment with chloroquine. Clinical failure was also higher amongst children treated with chloroquine than those treated with sulfadoxine-pyrimethamine alone or the combination. In children treated with chloroquine and sulfadoxine-pyrimethamine, parasite infection was found in 14% of participants at 28 days of follow-up. A number of children carried parasites predicted to be resistant to both chloroquine and sulfadoxine-pyrimethamine. However, these children were not significantly more likely to fail treatment.

**Strengths and limitations:** The trial took place in an area where there is, to date, little data on the efficacy of the different treatment regimens studied. The trial is also part of an ongoing series of other studies, which will ultimately allow researchers to detect changes in the efficacy of different treatments over time, providing information about parasite selection and the useful therapeutic life of particular treatments. However, the loss to follow-up in this trial was high (between 25 and 32%, depending on the treatment arm) due to logistical constraints around the time of the Gambian national election. It is not possible to tell if data from the participants lost to follow-up would have changed the overall conclusions.

**Contribution to the evidence:** This study provides additional data about the efficacy of chloroquine / sulfadoxine-pyrimethamine treatment in the Gambia, as compared to chloroquine or sulfadoxine-pyrimethamine alone. Prior evidence was not strong enough to confirm the advantage of chloroquine / sulfadoxine-pyrimethamine over chloroquine alone. The identification of parasites resistant to both chloroquine and sulfadoxine-pyrimethamine indicates that new combination drugs are needed in this part of Africa.

## INTRODUCTION

Malaria control in Africa has until recently relied heavily on chemotherapy with chloroquine (CQ), a cheap and safe antimalarial drug [1,2]. CQ remains widely distributed and readily available even in the most remote villages in sub-Saharan Africa. In the Gambia, each village with a population of 400 or more has a trained village health worker who is provided with chloroquine and other basic drugs [3]. Chloroquine-resistant strains of *Plasmodium falciparum,* first observed in East Africa in 1987, have now been reported in all countries of tropical Africa [4].

Studies between 1994 and 2000 found that 65–73% of Gambian children treated with chloroquine were parasitaemic at some point over 28 days of follow-up [5,6], demonstrating that as in much of Africa, CQ had ceased to be a satisfactory first-line treatment for uncomplicated P. falciparum malaria and an alternative was urgently required [7]. There have been a number of trials in sub-Saharan Africa to measure the efficacy, effectiveness, and impact on transmission of a variety of combination antimalarial regimens, including those incorporating a member of the artemisinin family [6,8–14]. Although the use of these newer combinations as first-line treatment for malaria is being adopted as policy in many African countries, interim solutions have been urgently sought by some prior to the full-scale introduction of artemisinin-based combination therapy.

In the Gambia, the combination of CQ and sulfadoxine-pyrimethamine (SP) has been shown to be a more effective symptomatic treatment than SP alone for malaria [8]. CQ/SP has therefore been used increasingly in the face of spreading resistance to CQ, and the combination was officially adopted as an affordable alternative frontline therapy in 2004. However, few reliable data are available on the efficacy of the combination CQ/SP in the Gambia, and although recent work has described the contribution of mutations in the CQ-resistance-associated loci *pfcrt* and *pfmdr1* to CQ treatment failure, to enhanced transmission to mosquitoes, and to an excess of severe malaria among rural Gambian children (15–17), there are no recent Gambian studies examining the prevalence of mutations associated with SP resistance, in the *pfdhfr* and *pfdhps* genes, nor of their effect on treatment outcome.

This paper reports the result of a randomised controlled trial conducted in 2001 to evaluate the efficacy of CQ/SP compared to SP or CQ alone. We also measured the pretreatment prevalence of parasites carrying resistance-associated alleles of four genes previously implicated in treatment failure of CQ and SP, and evaluate their contribution to therapeutic outcome.

## METHODS

### Participants

The study took place from September to December 2001 in Farafenni, a rural town on the north bank of the Gambia River. It is located on the Senegal border about 170 km from the Atlantic coast. The town has been the site for clinical trials to determine the efficacy of antimalarial drug combinations with gametocyte carriage and transmission as major endpoints since 1998 [6,10,11,13,14]. The climate is characteristic of the sub-Sahel with a short rainy season from mid-June to mid-October. Malaria is thus seasonal with most clinical episodes occurring during a limited period of 8 to 10 wk at the end of the rains.

Recruitment took place at the Maternal and Child Health clinic according to an established protocol [6]. Briefly, children 0.5–10 y of age living within 15–20 km radius of Farafenni who presented to the Maternal and Child Health clinic or the General Hospital with history of fever and/or current fever (axillary temperature, ≥37.5 °C) and other symptoms suggestive of acute malaria infection, a carriage of P. falciparum parasites at a density ranging between 500 and 250,000 parasites/μl of blood, and a packed cell volume (PCV) ≥20% were enrolled in the study after obtaining the free and informed consent of their parents or guardians. Excluded from the study were children with anaemia (PCV, <20%), any signs or symptoms of severe malaria, inability to take drugs orally or any evidence of chronic disease, malnutrition or any other acute infection, including non-falciparum malaria. If there was evidence of treatment with any antimalarial drug within the past 2 wk, either from notes on the clinic card carried by children under 5 y of age, or after questioning of the caregiver, the child was excluded. The study protocol was reviewed and approved by the Medical Research Council/Gambian Government Joint Ethics Committee, and the Ethics Committee of the London School of Hygiene and Tropical Medicine.

### Interventions

**Figure 1:**
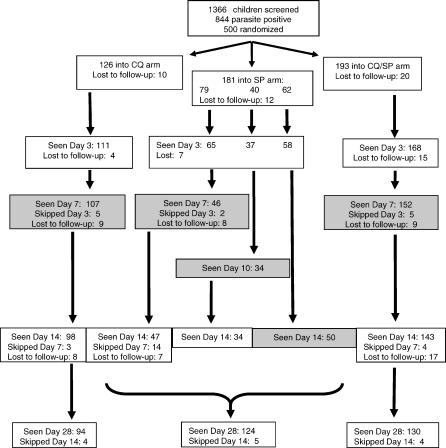
CONSORT Flowchart A total of 1,366 children was screened, and 500 were randomised. Children randomised to receive SP on Thursday or Friday (*N* = 79), were scheduled for standard follow-up and thus fulfilled the criteria for efficacy evaluation. Follow-up data are shown for these 79, but not for the other SP-treated children. Shaded boxes represent gametocyte screening days [18].

We performed a single-blind, randomised, controlled trial of oral treatment of uncomplicated falciparum malaria with CQ, SP, and the combination CQ/SP. A thorough clinical history (including demographic data) was taken, and a medical examination was performed by a study clinician on the day of screening, day 0. At screening and at each visit, body temperature was measured with a digital thermometer (Toshiba, Tokyo, Japan). The treatments used were CQ alone (25 mg of CQ base per kilogram of body weight over a 3-d period: 10 mg/kg on days 0 and 1; 5 mg/kg on day 2), SP alone given in a single dose (25 mg/kg sulphadoxine/1.25 mg/kg pyrimethamine), or the combination of CQ/SP. The group sizes were 130:180:200, respectively. This ratio was derived a priori as likely to ensure sufficient power for both efficacy and transmission endpoints of the trial, taking into account that children randomised to receive SP were further divided into three groups according to the day of the week on which they were recruited, as part of the accompanying study of post-treatment transmission [18]. These three SP-treated groups differed in their follow-up schedule, and only SP-treated patients recruited on Thursday or Friday received identical follow-up to children receiving CQ or CQ/SP. Therefore, only these patients are included in the analysis of clinical efficacy (Figure 1). Treatment allocation and prescription of the required medication were performed by a study clinician, and medicines were administered by a study nurse. Treatment was performed in a separate room but was not deliberately obscured from the view of the study team. Older children chewed or swallowed the tablets whole with water. For younger children, the tablets were crushed together in a cup and mixed with water before administration. Subsequent doses, for those in chloroquine groups, were supervised by trained field assistants in the patients' homes. All treated children were observed for 30 min, and any child who vomited was administered a replacement dose. Children who vomited both the initial and the repeat medication were excluded from the study and either given parenteral CQ, which at the time of the study was standard care in the Gambia for children with uncomplicated malaria who could not retain oral medication, or admitted to the paediatric ward of Farafenni Hospital.

In addition to the study drugs, oral paracetamol (10 mg/kg, three times daily) was given together with the study medication in the clinic, and additional doses (to last for 3 d) were given to parents/guardians for administration to their children at home.

### Objectives

The principal objective was to assess the efficacy of SP and CQ/SP for the treatment of uncomplicated falciparum malaria in children, compared to the efficacy of CQ monotherapy. A secondary objective was to estimate the prevalence of alleles of parasite genes associated with resistance to CQ and SP.

### Outcomes

The primary outcome was parasitaemia at any time during follow-up. Secondary outcomes were PCR-corrected group estimates of treatment failure, and clinical failure requiring rescue medication by day 28. Pretreatment parasite isolates from 161 patients were tested for the presence of resistance-associated genetic markers.

### Sample Size

The sample size required in each treatment group was estimated with transmission endpoints in mind. We expected to enroll approximately 500 patients, based on data from 1998–2000. In our previous studies, the observed prevalence of gametocyte carriage 7 d after treatment with SP was >70% (excluding those who had gametocytes on day 0), and 40% after treatment with CQ [13]. We therefore expected that the prevalence of gametocytes after SP/ CQ would be at least 28% (i.e., 40% × 70%). Based on these figures, and to ensure that at least 30 mosquito feeds were performed in each treatment group, we set out to enroll (a) 200 subjects in the CQ + SP group, (b) 50, 60, and 70 subjects in the three groups receiving SP and being screened for gametocytes on days 7, 10, and 14, respectively, and (c) 130 subjects in the CQ group. All CQ- and CQ/SP-treated children were to be screened for gametocytes on day 7 only. This sample size was expected to provide sufficient power at the 90% confidence level to detect a 10-fold difference in infectiousness at day 7 between gametocyte-positive children treated with SP alone, and those treated with SP + CQ [18]. This was not expected to provide sufficient power to detect any difference in parasitological efficacy between SP and CQ/SP, which was estimated a priori to be approximately 90% for both regimens.

### Randomisation—Sequence Generation

Eligible patients were randomly assigned to treatment using a predetermined computer generated randomisation list. The list was generated by a statistician not otherwise engaged in the study.

### Randomisation—Allocation Concealment

Allocation was not concealed, but taken from a list with 550 allocations on it, in the order of recruitment. Access to this list was restricted.

### Randomisation—Implementation

Patients underwent initial clinical and parasitological screening to confirm a diagnosis of malaria before being asked for informed consent by a field worker able to speak Wolof, Mandinka, and Fulani. This person did not have access to the allocation list. If consent was given, a clinical examination, treatment allocation, and prescription of the required medication were performed in a separate room by a study clinician who had access to the randomisation list.

### Randomisation—Blinding

Only the study clinician responsible for initial recruitment and treatment, and the field assistants visiting the children during follow-up, had knowledge of drug allocations. Field assistants did not have access to the full allocation list, but knew the treatment group of children in their care. All laboratory staff, microscopists, entomologists, and the principal investigator were blinded to treatment allocations.

### Post-Treatment Follow-Up

Field assistants visited children at home on days 1 and 2 to supervise treatment (to those in CQ groups) and on days 3, 14, and 28 to enquire from the caretaker about the child's condition of health and to collect finger prick samples that provided thick blood films for microscopy and filter paper blood spots for parasite typing by PCR. If on any of these visits the caretaker was concerned about the child's health, he or she was asked to attend the clinic as soon as possible. Parents/guardians were also requested to bring their children to the Maternal and Child Health clinic at any time in the event of clinical aggravation. If a parent or guardian reported that a child was unwell, the child was examined by a study clinician. If the child had parasitaemia and fever (axillary temperature, ≥37.5 °C) or recent history of fever and did not have other conditions that could explain the symptoms, and fewer than 29 d had elapsed since recruitment, the child was considered a clinical treatment failure and given rescue treatment as follows: those in the CQ treatment group received SP, and those in the other two groups received a standard course of oral quinine. Children presenting with persistent symptoms of malaria within 3 d of enrollment with >90% reduction in parasitaemia were not considered to be clinical treatment failures, and did not receive rescue treatment. They were monitored until complete recovery.

On day 7 after treatment, the children were collected from their homes and taken to the Medical Research Council Field Station in Farafenni where they were clinically examined and finger prick blood samples were obtained for thick blood film preparation and PCV estimation. The blood films were stained with Field's stain and read immediately for malaria parasites. Some children identified as gametocyte carriers at this time contributed to transmission experiments described elsewhere [18].

### Blood Sampling and Laboratory Measurements

At screening and at day 3, 7, 14, and 28 follow-up visits, blood samples were obtained by finger prick for thick blood smears for malaria microscopy. At day 7, or any time that the child had symptoms consistent with clinical malaria, two thick smears were made; the first was stained with Field's stain and read immediately. The second was dried overnight, stained with Giemsa, and read later by two experienced laboratory assistants to provide definitive parasite counts. One hundred high-power fields were read before a slide was declared negative. At screening and at day 7, blood samples were collected in heparinised capillary tubes and spun using a micro-haematocrit centrifuge (Hawksley, West Sussex, United Kingdom) for PCV determination. Staff performing all laboratory investigations were blinded to treatment regimens.

### Molecular Genotyping

P. falciparum genotypes circulating in children with detectable asexual parasitaemia during posttreatment follow-up were compared with those present in the same child prior to treatment. DNA was extracted from dried blood spots using a Chelex-based method [19]. Alleles of the polymorphic locus *pfmsp2* were compared between pretreatment and posttreatment parasite isolates by PCR [11,20]. The procedure of Cattamanchi et al. [21] was followed, in that indeterminate samples in which a majority of novel bands appeared in the posttreatment infection were scored as new infections.

Resistance-associated loci encoding amino acid positions 72–76 in *pfcrt* (sensitive allele, Cys-Val-Met-Asn-Lys [CVMNK]; resistant allele, Cys-Val-Ile-Glu-Thr [CVIET]), position 86 in *pfmdr1* (resistant allele, Asn [N]; sensitive allele, Tyr [Y]), positions 51, 59, and 108 in *pfdhfr* (resistant alleles, Ile, Arg, and Asn [I, R, N], respectively; sensitive alleles, Asn, Cys, and Ser [N, C, S], respectively), and positions 437 and 540 in *pfdhps* (sensitive alleles, Ala and Lys [A, K], respectively; resistant alleles, Gly and Glu [G, E], respectively) were identified as previously described [15,22,23]. Briefly, fluorescent-labeled oligonucleotide probes specific for each allele of interest were hybridised to PCR products spotted in 12 × 8 arrays on nylon membranes, and hybridisation signals were detected by chemiluminescence.

### Statistical Methods

All data were double entered and verified using Epi-Info, version 6 (Centers for Disease Control and Prevention, Atlanta, Georgia, United States) and transferred to Stata 7.0 (Stata Corporation, College Station, Texas, United States) for statistical analysis. All children with follow-up data were included in the primary analysis. Any child presenting with danger signs on days 0–3 in the presence of malaria parasites or fever/history of fever plus parasitaemia any time from days 4 to 28 was given rescue medication and treated as a clinical therapeutic failure. Proportions were compared using the χ^2^ statistic for parasitological data (denominators >50 in each case) or using Fisher exact test for clinical failure data and molecular genotyping data, because in both these analyses expected proportions in some cells of 2 × 2 tables were ≤5. Asexual parasite density was compared between groups using the ratio of arithmetic means. This was done by fitting generalized linear models with the negative binomial distribution family and logarithmic link function of the (arithmetic) mean parasite density as previously described [11]. Confidence intervals around projected relative risk estimates were calculated from the variance of the log relative risk as described [24], after modification to allow for each risk being the product of two proportions.

## RESULTS

### Recruitment and Participant Flow

A total of 1,368 children was screened. Two records were without parasitaemia data and were therefore excluded from further analysis. Of the 1,366 children with complete information on malaria infection, 844 (61.8%) were positive for malaria parasites according to microscopy at the recruiting clinic, and were therefore eligible for enrolment into the study. Of these, 500 were enrolled into the trial; 126, 181, and 193 in the CQ alone, SP alone, and CQ/SP groups, respectively. The most common reasons for ineligibility for enrollment were low density parasitaemia (<500/μl of blood) (*n* = 114), refusal to give parental consent (*n* = 40), anemia (PCV, <20%) (*n* = 34), residence outside the clinic catchment area (*n* = 24), high density parasitaemia (≥250,000 parasites/μl of blood), and/or other severe signs of malaria (*n* = 18) and presence of concomitant diseases and other reasons (*n* = 51). A trial profile is shown in Figure 1. Loss to follow-up by day 28 was 25.4, 31.5, and 32.6% in the CQ, SP, and CQ/SP treatment groups, respectively.

### Baseline Data

There were no differences in baseline demographic characteristics or malaria infection between the treatment groups (Table 1).

**Table 1:**
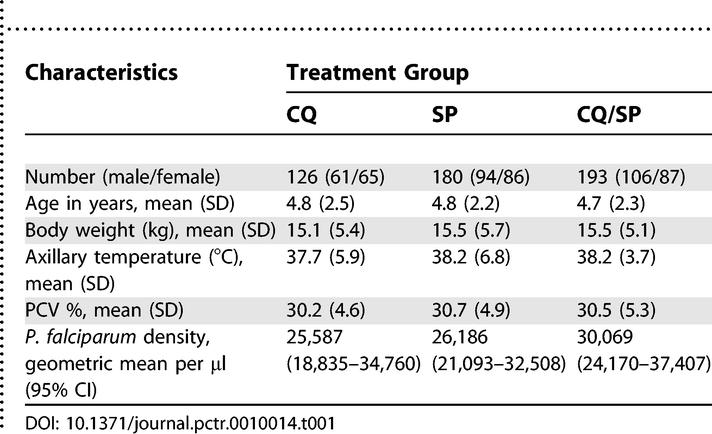
Baseline Demographic and Clinical Characteristics of Patients Randomised to CQ Alone, SP Alone, or CQ/SP Treatment

### Outcomes and Estimation

#### Clinical outcomes and adverse advents.

The frequency of posttreatment, parasitologically confirmed clinical malaria (clinical failure) among evaluable study patients in each treatment group is shown in Table 2. By day 28, as expected from previous studies, more children in the CQ treatment group had experienced an episode of clinical malaria posttreatment than in either of the other groups. There was weak evidence that clinical failure was more common among SP-treated children than among those receiving CQ/SP (OR, 2.68; 95% CI, 0.764–9.34). This was apparently due to persistence of symptoms during the first 3 d (Table 2), consistent with the findings of Bojang et al. [8]. However, clinical endpoints were not the main focus of the trial, and we did not collect sufficient data to make a valid comparison with earlier results [8]. There were no serious adverse events reported during follow-up, and no deaths among the 500 recruited participants during the study period.

**Table 2:**
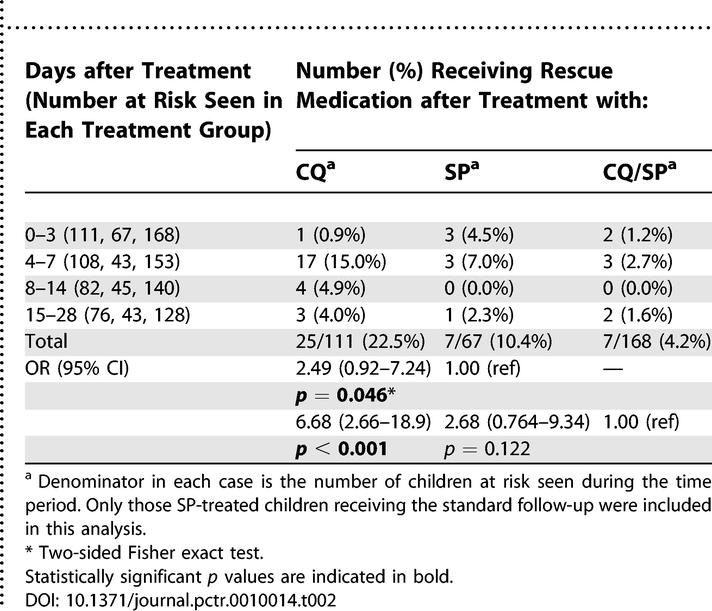
Clinical Failure Rates in Children Treated with CQ, SP, or CQ/SP for Uncomplicated P. falciparum Malaria

#### Parasitological outcomes.

**Figure 2:**
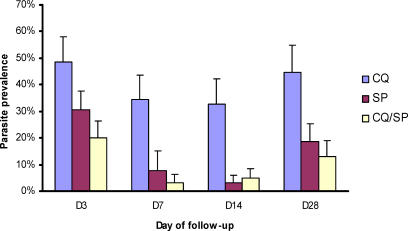
Point Prevalence of Plasmodium falciparum Asexual Parasitaemia 3, 7, 14, and 28 Days after Treatment of Children with CQ, SP, or CQ/SP Error bars represent the upper 95% confidence limit of the proportion. Denominators for these data are (for CQ, SP, and CQ/SP groups, respectively): day 3, 111, 160, 168; day 7, 107, 52, 152; day 14, 98, 134, 143; and day 28, 94, 124, 130.

The point prevalence of parasitological treatment failure for each treatment group at each day of follow-up is shown in Figure 2. This is a per protocol analysis. At each time point, parasitological failures were most common among CQ-treated children, with an uncorrected cumulative treatment failure rate over 28 d of 60.3% for CQ, compared to 17.6% for SP (OR, 0.106; 95% CI, 0.057–0.194; *p* < 0.001) and 13.9% for CQ/SP (OR compared to CQ, 0.140; 95% CI, 0.078–0.250; *p* < 0.001). To compare the parasitological outcomes of each group in more detail, the ratio between arithmetic mean parasite densities at each day of active follow-up was calculated, and the statistical significance of this difference tested by negative binomial regression, including those with zero parasites (Table 3). We have previously used this method to compare gametocyte densities among treatment groups [11,14]. We found that the parasitological efficacy of SP was significantly higher than that of CQ at days 7, 14, and 28, and that of CQ/SP was significantly higher than that of CQ at days 3, 7, and 14 (Table 3). No significant difference in mean parasite density was found between the SP and CQ/SP groups at any point (data not shown).

**Table 3:**
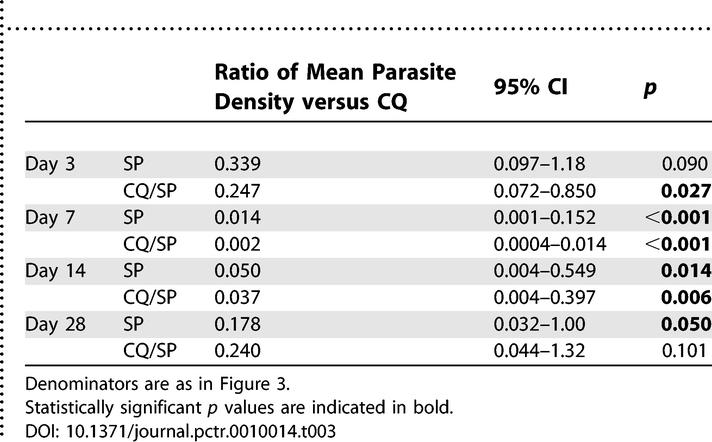
Parasitological Benefit of SP and CQ/SP Treatment Compared to CQ Treatment at Each Point of Follow-Up

**Figure 3:**
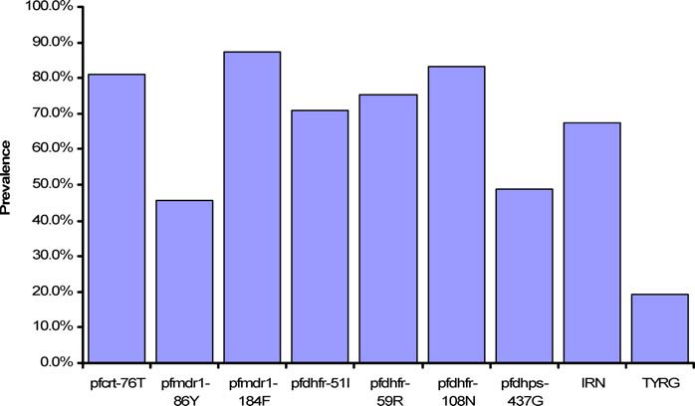
Prevalence of Resistance-Associated Loci among 90 Pretreatment Parasite Isolates Genotypes at seven loci in four P. falciparum genes were determined, as was the prevalence of the *pfdhfr* triple-mutant IRN, and of the putative multidrug-resistant genotype TYRG.

### Correction of Parasitological Failure Rates by Msp2 Genotyping

Alleles of *msp2* in pretreatment DNA samples were compared to those in posttreatment DNA samples to distinguish recrudescent parasites from parasites newly emergent from the liver during follow-up, as previously described [11]. PCR was performed for paired isolates from 20 patients with posttreatment parasitaemia from each treatment group, and interpretable data obtained for 16, 15, and 17 pairs from the CQ, SP, and CQ/SP groups, respectively. Results are tabulated in Table 4, and used to estimate the true failure rate in each group by extrapolation. We found a corrected failure rate for CQ of 30.2%, for SP of 6.06%, and for CQ/SP of 3.94%. Using these estimates, we also projected a “virtual” relative risk of posttreatment recrudescence in each treatment group (Table 4). Both treatment groups containing SP were substantially better than CQ monotherapy in this analysis, whereas there was no significant benefit identified for CQ/SP over SP alone.

**Table 4:**
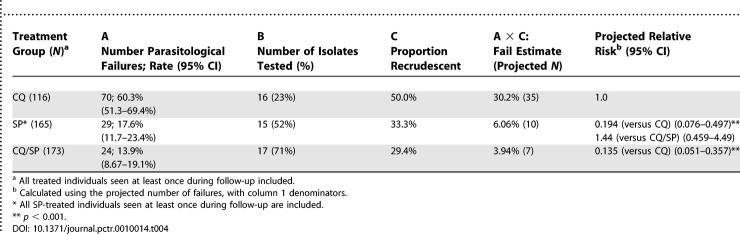
Cumulative 28-d Parasitological Failure Rates Corrected by *msp2* Genotyping

### Baseline Prevalence of Resistance-Associated Alleles

The prevalence of drug resistance-associated mutations at seven loci in four genes was measured among 90 day 0 samples randomly selected from among all trial participants (Figure 3). The prevalence of the *pfdhfr* triple mutant 51-I, 59-R, 108-N (IRN) was also measured, as was prevalence of the combination genotype *pfcrt-*76T, *pfmdr1*-86Y, *pfdhfr-*59R, and *pfdhps-*437G (TYRG), a possible multidrug-resistant genotype. Combination genotypes IRN and TYRG were assigned to infections in which each allele was present. However, in some such individuals, wild-type alleles were also present at some of the loci in the combination, and so it is possible that IRN or TYRG did not occur as a single haplotype. Of the 60 patients designated as IRN, 49 (81.7%) unequivocally harboured true triple-mutant haplotypes, whereas the other 11 patients were of mixed genotype at two or three of the *dhfr* resistance-associated loci. Of the 17 patients who harboured parasites carrying the mutations *pfcrt-*76T, *pfmdr1*-86Y, *pfdhfr-*59R, and *pfdhps-*437G, the resistant allele alone was detected at three or four of these loci in 11 cases (64.7%). Thus, we are certain that parasites of the haplotype TYRG must occur in these infections. Mixed sensitive and resistant alleles were detected at two loci in four further isolates, and at three and all four loci in the remaining two isolates, respectively. Thus, in these latter six isolates, the haplotype TYRG may occur, but this is not certain. All were retained in subsequent analysis of associations with treatment failure.

### Resistance-Associated Alleles and Treatment Failures

The risk of treatment failure associated with resistant genotypes at presentation was investigated by examining resistance-associated loci in pretreatment isolates from 100 patients who subsequently failed treatment. These comprised 44 children who received CQ, 31 who received SP, and 25 who received CQ/SP, of which four, three, and three children, respectively, required rescue treatment at the time of failure due to recurrent or persistent clinical signs of malaria. The prevalence of each marker in these pretreatment samples was compared to the prevalence among 61 isolates from children who were successfully treated. These children, 7, 22, and 32, respectively, from the CQ, SP, and CQ/SP groups, were among the 90 randomly selected isolates described in Figure 3. Because 29 of these children subsequently failed treatment, only 61 were used for the purposes of estimating the odds of failure associated with each pretreatment genotype. A major purpose of these exploratory analyses was to identify a haplotype adequately representing “multidrug resistance” that could be defined at a single locus for each gene and so did not require using data at all seven loci. Estimates of the association between treatment failure and both single locus and selected multilocus genotypes is presented in Table 5. The only statistically significant association with treatment failure for any single locus was in the SP treatment group, where children presenting with the *pfdhps-*437G allele were more likely to fail treatment than those carrying the wild-type 437A allele (Table 5). Other weak associations could be discerned in the data, but given the multiple testing performed, these findings were not considered reliable. The combined dual locus genotype TY defined at *pfcrt-*76 and *pfmdr1-*86, previously associated with CQ treatment failure in this population [15], appeared to associate with treatment failure in the SP group only. The combined dual-locus genotype RG defined at *pfdhfr-*59 and *pfdhps-*437 was strongly associated with treatment failure, also in the SP group only, suggesting that the combination of these two alleles comprise the dominant SP-resistant haplotype in this study area. The four locus genotype combining both of these two allele genotypes, TYRG, was also strongly associated with treatment failure only in the SP treatment group (Table 5).

**Table 5:**
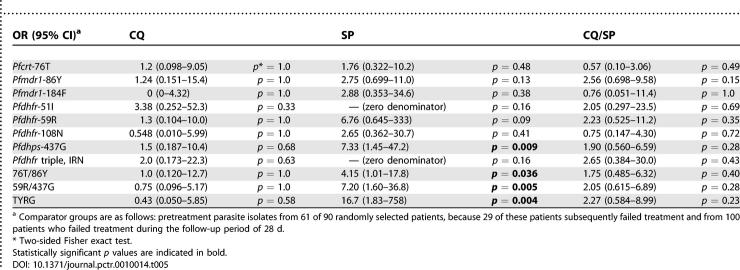
Odds of Subsequent Treatment Failure for Each Single-Locus Genotype, the *pfdhfr* Triple Mutant, and the Four-Locus Genotypes TFRG and TYRG at Day 0, in Each Treatment Group

## DISCUSSION

### Interpretation

In this study, we have examined the efficacy of the combination CQ/SP for treating uncomplicated falciparum malaria in Gambian children, compared to either CQ or SP alone. Pairwise comparisons of parasitological outcomes between regimens at each point of follow-up demonstrated a sustained benefit of treatment with SP or CQ/SP compared to CQ alone, but no difference in efficacy was found between the two SP-containing treatment groups at any time point. Cumulative failure rates, after correction by *msp2* genotyping to estimate rates of recrudescence, did not differ between the SP- and CQ/SP-treated groups of patients. The clinical failure rate of CQ monotherapy over 28 d was 22.6% in this study, and thus CQ is no longer useful as first-line treatment for malaria in children. The combination CQ/SP is an efficacious treatment for uncomplicated malaria in Gambian children, but the high prevalence of CQ-resistant parasites coupled with a small but measurable SP failure rate strongly suggest that this level of efficacy is unsustainable in our study area. These data were collected in late 2001, and continued use of CQ/SP as first-line treatment may have further eroded the efficacy of this combination since that time. However, subsequent studies show that crude parasitological efficacy for CQ/SP in Farafenni has remained good at 88% in 2002 [11] and 86% in 2003 (P. Milligan and S. Dunyo, unpublished data). CQ/SP has been the first-line antimalarial regimen in the Gambia since 2004.

In 1995, Bojang et al. [8] measured the parasitological efficacy of SP and CQ/SP in children aged 1–10 y presenting to health facilities at Sibanor and Basse, the Gambia. These authors found the cumulative (uncorrected) parasitological failure rate at day 28 was 10% for SP (15 of 150) and 5% (7 of 141) for CQ/SP. These compare with uncorrected rates of 17.6% (29 of 165) and 13.9% (24 of 173) in the present study. Assuming the methodologies used are compatible, this suggests that children treated with SP in 2001 had a relative risk of parasitological failure of 1.31 (*p* = 0.053) compared to children treated with SP in 1995. Children treated with CQ/SP in 2001 had a relative risk of failure of 1.47 (*p* = 0.009). This exploratory comparison does suggest that the efficacy of both SP and CQ/SP has diminished in the 5-y period between these two studies. Bojang et al. did find that CQ/SP provided more rapid resolution of malaria symptoms than treatment with SP alone, but this was not tested in our study.

We used *msp2* genotyping in a subset of parasitological treatment failures to estimate the recrudescence rate in each treatment group. This method is prone to underestimate the true number of recrudescent infections in areas of stable transmission, because the PCR method will not pick up minority genotypes that may be present in the pretreatment sample at low abundance. A multiplicity of infection is seen in most infected patients in sub-Saharan Africa, and, among children enrolled in our study, there were on average four genotypes per infection prior to treatment (R. Ord and C. J. Sutherland, unpublished data). Minority genotypes that are resistant may flourish under drug selection and become dominant during follow-up. These will appear to be a “new” infection, but are actually recrudescent [20]. Thus PCR correction must be interpreted carefully, and for this reason we prefer to project a comparative estimate of the true failure rate among the treatment groups, rather than assign each posttreatment parasitaemia its own status as a recrudescent or a new infection.

In our study area, addition of SP to CQ provided a therapeutic efficacy of over 90% after PCR correction [6; this study], and is currently the recommended regimen in the Gambia. SP alone also provides parasitological efficacy of approximately 90% over 28 d [9; this study]. We were therefore surprised to find high prevalences of both the *pfdhfr* “IRN” triple mutant and the *pfdhps-*437G allele in our pretreatment population. The absence of the *pfdhfr-*164L and *pfdhps-*540E mutations in this study area (R. Hallett, R. Ord, and A. Randall, unpublished data) may explain why a reasonable level of SP efficacy is retained, although a lack of correlation between mutations and outcomes in individual patients is frequently observed in clinical trials of antimalarial treatment [25,26]. Nevertheless, the single marker *pfdhps-*437G was strongly associated with SP treatment failure in univariate analyses (Table 5).

### Generalizability

Our study suffered from a high dropout rate due to logistic constraints exacerbated by national elections in the Gambia in October 2001, which meant that many staff and participants traveled away from the study area. Nevertheless, estimates of efficacy were consistent with other studies in the area in 1998 and 1999 (SP) [13], in 1998 and 2000 (CQ) [6,13], and in 2002 and 2003 (CQ/SP) [11]. Therefore, finding that SP and CQ/SP are efficacious treatments despite the common occurrence of resistance-associated genotypes in four genes of interest is likely to be generally true. Multidrug-resistant parasite genotypes have recently been described in a single case report from South Africa [27], but have not been widely investigated in African parasite populations, nor has the impact of such genotypes on treatment outcomes been measured in clinical trials. We found that P. falciparum parasites with the multidrug-resistant genotype TYRG, defined at the four loci *pfcrt-*76, *pfmdr1-*86, *pfdhfr*-59, and *pfdhps-*437 respectively, are relatively common among Gambian children presenting with uncomplicated malaria. The TYRG genotype was associated with therapeutic failure after treatment with SP, but not with the combination CQ/SP. Unexpectedly, there was also a weak association between SP treatment failure and the carriage of CQ resistance-associated alleles of *pfcrt* and *pfmdr1*. This may reflect the relatively low power of our study, and the exploratory nature of our analyses, which did not correct for multiple testing. Furthermore, the high prevalence of *pfcrt-*76T in the parasite population is likely to have masked associations between CQ-resistance loci and treatment outcomes. Nevertheless, these preliminary results do demonstrate the need for carefully designed studies to measure the contribution of multidrug-resistant parasites to inadequate treatment of uncomplicated malaria in African children as combination treatments become more widely deployed. Inadequate treatment is likely to increase risk of progression to severe disease, particularly severe malarial anaemia [17], and thus short-term gains in terms of improved treatment efficacy achieved with combinations such as CQ/SP and amodiaquine/SP may be quickly eroded if multidrug-resistant genotypes enjoy a selective advantage in the treated host. In the accompanying paper, we investigate the nature of that selective advantage in Anopheles gambiae mosquitoes experimentally fed on gametocytes from children who had received the CQ/SP combination [18]. The results suggest that children harbouring multidrug-resistant parasites are significantly more infectious to mosquitoes than other CQ/SP-treated children.

### Overall Evidence

A recent systematic review found a poor evidence base for the therapeutic efficacy of CQ/SP [28], yet this drug combination has been first-line therapy for uncomplicated malaria in the Gambia since 2004. We have identified multidrug-resistant parasite genotypes of P. falciparum carrying alleles implicated in resistance to both CQ and SP, and found these to be common in our study area in 2001. Nevertheless, these parasites did not substantially challenge the therapeutic efficacy of SP or CQ/SP in this study, which were found to have 82.4 and 86.1% efficacy against recurrent parasitaemia, respectively. The prevalence of these genotypes suggests they are advantageous to the parasite. Therefore, continued use of CQ/SP may favour an increase in the prevalence of SP resistance-associated alleles, and should the absent *pfdhps-*540E mutation be introduced to the population the efficacy of the combination may then drastically decline [25]. The risk of this occurring is heightened by the prevalence of CQ-R parasites in the Gambia [15–17; this study], such that SP has been added to a drug that is already failing. It has been demonstrated that the addition of artesunate to CQ produces a poor therapeutic combination [6]. A possible interim solution in the Gambia for the period leading up to implementation of ACTs may be amodiaquine plus SP, a combination found to work very well in other settings where CQ resistance is high but amodiaquine remains efficacious [29].

## SUPPORTING INFORMATION

CONSORT Checklist Click here for additional data file. (50 KB DOC)

Trial Protocol Click here for additional data file. (88 KB DOC)

## Abbreviations

CI: confidence interval
CQ: chloroquine
OR: odds ratio
PCV: packed cell volume
SP: sulphadoxine-pyrimethamine

## References

[pctr-0010014-b001] World Health Organization [WHO] (1993). Implementation of the global malaria control strategy. Technical Report Series Number 839.

[pctr-0010014-b002] World Health Organization [WHO], United Nations Children's Fund [UNICEF] (2003). Africa Malaria Report 2003.

[pctr-0010014-b003] Greenwood BM, Bradley AK, Byass P, Greenwood AM, Menon A (1990). Evaluation of a primary health care programme in The Gambia. II. Its impact on mortality and morbidity in young children. J Trop Med Hyg.

[pctr-0010014-b004] Wellems TE, Plowe CV (2001). Chloroquine-resistant malaria. J Infect Dis.

[pctr-0010014-b005] Müller O, Boele van Hensbroek M, Jaffa S, Drakeley C, Okorie C (1996). A randomized trial of chloroquine, amodiaquine and pyrimethamine-sulfadoxine in Gambian children with uncomplicated malaria. Trop Med Int Health.

[pctr-0010014-b006] Sutherland CJ, Drakeley CJ, Obisike U, Coleman R, Jawara M (2003). The addition of artesunate to chloroquine for treatment of Plasmodium falciparum malaria in Gambian children delays, but does not prevent treatment failure. Am J Trop Med Hyg.

[pctr-0010014-b007] White NJ, Nosten F, Looareesuwan S, Watkins WM, Marsh K (1999). Averting a malaria disaster. Lancet.

[pctr-0010014-b008] Bojang KA, Schneider G, Forck S, Obaro SK, Jaffar S (1996). A trial of Fansidar^®^ plus chloroquine or Fansidar^®^ alone for the treatment of uncomplicated malaria in Gambian children. Trans R Soc Trop Med Hyg.

[pctr-0010014-b009] von Seidlein L, Bojang K, Jones P, Jaffar S, Pinder M (1998). A randomized controlled trial of artemether/benflumetol, a new antimalarial and pyrimethamine/sulfadoxine in the treatment of uncomplicated falciparum malaria in African children. Am J Trop Med Hyg.

[pctr-0010014-b010] von Seidlein L, Milligan P, Pinder M, Bojang K, Anyalebechi C (2000). Efficacy of artesunate plus pyrimethamine-sulphadoxine for uncomplicated malaria in Gambian children: a double-blind, randomised, controlled trial. Lancet.

[pctr-0010014-b011] Sutherland CJ, Ord R, Dunyo S, Jawara M, Drakeley CJ (2005). Reduction of malaria transmission to Anopheles mosquitoes with a six-dose regimen of Co-Artemether. PLoS Med.

[pctr-0010014-b012] Mutabingwa TK, Anthony D, Heller A, Hallett R, Ahmed J (2005). Amodiaquine alone, amodiaquine+sulfadoxine-pyrimethamine, amodiaquine+artesunate, and artemether-lumefantrine for outpatient treatment of malaria in Tanzanian children: a four-arm randomised effectiveness trial. Lancet.

[pctr-0010014-b013] Targett G, Drakeley C, Jawara M, von Seidlein L, Coleman R (2001). Artesunate reduces but does not prevent posttreatment transmission of Plasmodium falciparum to Anopheles gambiae. J Infect Dis.

[pctr-0010014-b014] Drakeley CJ, Jawara M, Targett GA, Walraven G, Obisike U (2004). Addition of artesunate to chloroquine for treatment of Plasmodium falciparum malaria in Gambian children causes a significant but short-lived reduction in infectiousness for mosquitoes. Trop Med Int Health.

[pctr-0010014-b015] Sutherland CJ, Alloueche A, Curtis J, Drakeley CJ, Ord R (2002). Gambian children successfully treated with chloroquine can harbour and transmit Plasmodium falciparum gametocytes carrying resistance genes. Am J Trop Med Hyg.

[pctr-0010014-b016] Hallett RL, Sutherland CJ, Alexander N, Ord R, Jawara M (2004). Combination therapy counteracts the enhanced transmission of drug-resistant malaria parasites to mosquitoes. Antimicrob Agents Chemother.

[pctr-0010014-b017] Meerman L, Ord R, Teun Bousema J, van Niekerk M, Osman E (2005). Carriage of chloroquine-resistant parasites and delay of effective treatment increase the risk of severe malaria in Gambian children. J Infect Dis.

[pctr-0010014-b018] Hallett RL, Dunyo S, Ord R, Jawara M, Pinder M (2006). Chloroquine/Sulphadoxine-Pyrimethamine for Gambian Children with malaria: Transmission to Mosquitoes of Multidrug-Resistant Plasmodium falciparum. PLoS Clin Trials.

[pctr-0010014-b019] Plowe CV, Djimde A, Bouare M, Doumbo O, Wellems TE (1995). Pyrimethamine and proguanil resistance-conferring mutations in Plasmodium falciparum dihydrofolate reductase: polymerase chain reaction methods for surveillance in Africa. Am J Trop Med Hyg.

[pctr-0010014-b020] Snounou G, Beck H-P (1998). The use of PCR genotyping in the assessment of recrudescence or reinfection after antimalarial drug treatment. Parasitol Today.

[pctr-0010014-b021] Cattamanchi A, Kyabayinze D, Hubbard A, Rosenthal PJ, Dorsey G (2003). Distinguishing recrudescence from reinfection in a longitudinal antimalarial drug efficacy study: comparison of results based on genotyping msp-1, msp-2 and glurp. Am J Trop Med Hyg.

[pctr-0010014-b022] Pearce RJ, Drakeley C, Chandramohan D, Mosha F, Roper C (2003). Molecular determination of point mutation haplotypes in the dihydrofolate reductase and dihydropteroate synthase of Plasmodium falciparum in three districts of northern Tanzania. Antimicrob Agent Chemother.

[pctr-0010014-b023] Cissé B, Sokhna C, Boulanger D, Milet J, Bâ EH (2006). Seasonal intermittent preventive treatment with artesunate and sulfadoxine pyrimethamine reduces the burden of malaria in Senegalese children. Lancet.

[pctr-0010014-b024] Kahn H, Sempos C (1989). Statistical methods in epidemiology.

[pctr-0010014-b025] Dorsey G, Dokomajilar C, Kiggundu M, Staedke SG, Kamya MR (2004). Principal role of dihydropteroate synthase mutations in mediating resistance to sulfadoxine-pyrimethamine in single-drug and combination therapy of uncomplicated malaria in Uganda. Am J Trop Med Hyg.

[pctr-0010014-b026] Mendez F, Munoz A, Carrasquilla G, Jurado D, Arevalo-Herrera M (2002). Determinants of treatment response to sulfadoxine-pyrimethamine and subsequent transmission potential in falciparum malaria. Am J Epidemiol.

[pctr-0010014-b027] Schwab U, Alloueche A, Doherty JF (2005). Multidrug-resistant malaria from South Africa. Clin Infect Dis.

[pctr-0010014-b028] McIntosh HM, Jones KL (2005). Chloroquine or amodiaquine combined with sulfadoxine-pyrimethamine for treating uncomplicated malaria. Cochrane Database Syst Rev.

[pctr-0010014-b029] Staedke SG, Mpimbaza A, Kamya MR, Nzarubara BK, Dorsey G (2004). Combination treatments for uncomplicated falciparum malaria in Kampala, Uganda: randomised clinical trial. Lancet.

